# 循环浆细胞在接受VRD方案诱导治疗的初诊多发性骨髓瘤中的预后价值

**DOI:** 10.3760/cma.j.cn121090-20241209-00549

**Published:** 2025-09

**Authors:** 若茹 刘, 叶 姚, 媛媛 金, 露 刘, 青林 史, 旭星 沈, 丽娟 陈

**Affiliations:** 南京医科大学第一附属医院，江苏省人民医院血液科，南京 210029 Department of Hematology, the First Affiliated Hospital with Nanjing Medical University, Jiangsu Province Hospital, Nanjing 210029, China

**Keywords:** 多发性骨髓瘤, 浆细胞, 流式细胞学, 预后, 循环浆细胞, Multiple myeloma, Plasma cells, Flow cytometry, Prognosis, Circulating plasma cells

## Abstract

**目的:**

探讨循环浆细胞（CPC）在接受VRD方案（硼替佐米+来那度胺+地塞米松）诱导治疗的初诊多发性骨髓瘤（NDMM）患者中的预后价值。

**方法:**

回顾性分析2019年1月至2024年3月在江苏省人民医院血液科接受VRD方案诱导治疗的152例NDMM患者的临床资料，比较CPC比例高、低组患者的临床特征、疗效及预后。分析CPC阳性组、CPC转阴组和CPC阴性组患者的预后。

**结果:**

本研究共纳入NDMM患者152例，其中男76例，女76例，中位发病年龄62（40～77）岁。与CPC比例<0.105％组相比，CPC比例≥0.105％组患者的ISS分期Ⅲ期比例（*P*<0.001）、R-ISS分期Ⅲ期比例（*P*＝0.023）、HGB≤100 g/L比例（*P*＝0.015）、β_2_-微球蛋白≥3.5 g/L比例（*P*<0.001）较高，中位无进展生存（PFS）期（24个月对52个月，*P*<0.001）及中位总生存（OS）期（52个月对未达到，*P*＝0.005）较短。CPC阴性组患者较CPC转阴组、CPC阳性组患者有更长的中位PFS期（未达到对41个月对19个月，*P*<0.001）和中位OS期（未达到对未达到对26个月，*P*<0.001）。多因素分析显示，影响患者PFS的独立危险因素包括CPC比例≥0.105％（*HR*＝3.79，95％*CI*：1.95～7.38，*P*<0.001）、诱导治疗后CPC阳性（*HR*＝3.54，95％*CI*：1.41～8.87，*P*＝0.007）和细胞遗传学高危（*HR*＝3.69，95％*CI*：1.85～7.37，*P*<0.001），影响患者OS的独立危险因素包括CPC比例≥0.105％（*HR*＝3.50，95％*CI*：1.29～9.48，*P*＝0.014）和诱导治疗后CPC阳性（*HR*＝4.12，95％*CI*：1.13～15.03，*P*＝0.032）。

**结论:**

CPC高表达的NDMM患者预后较差，CPC水平是NDMM患者的独立预后因素。

## Abstract (en)

**Objective:**

To investigate the prognostic value of circulating plasma cell（CPC）in patients with newly diagnosed multiple myeloma（NDMM）undergoing induction therapy with bortezomib, lenalidomide, and dexamethasone（VRD）regimen.

**Methods:**

This study retrospectively analyzed clinical data of 152 patients with NDMM treated with the VRD regimen as induction therapy in the Hematology Department of Jiangsu Provincial People's Hospital from January 2019 to March 2024. The clinical characteristics, efficacy, and prognosis of patients with high and low CPC proportions are compared. The prognosis of patients in the CPC-positive group, CPC-negative conversion group, and CPC-negative group was analyzed.

**Results:**

This study included 152 patients with NDMM, comprising 76 males and 76 females, with a median age at onset of 62（40–77）years. Compared with the group with CPC proportion of <0.105％, patients with CPC proportion of ≥0.105％ demonstrated a higher proportion of International Staging System（ISS）stage Ⅲ（*P*<0.001）, Revised ISS stage Ⅲ（*P*＝0.023）, HGB≤100 g/L（*P*＝0.015）, β_2_-microglobulin ≥3.5 g/L（*P*<0.001）, shorter median progression-free survival（PFS）period（24 months *vs* 52 months, *P*<0.001）, and shorter median overall survival（OS）period（52 months *vs* not achieved, *P*＝0.005）. Patients in the CPC-negative group demonstrated a longer median PFS period（not reached *vs* 41 months *vs* 19 months, *P*<0.001）and median OS period（not reached *vs* not reached *vs* 26 months, *P*<0.001）compared with patients in the CPC-negative conversion group and CPC-positive group. Multivariate analysis revealed CPC proportion of ≥0.105％（*HR*＝3.79, 95％ *CI*: 1.95–7.38, *P*<0.001）, positive CPC after induction therapy（*HR*＝3.54, 95％ *CI*: 1.41–8.87, *P*＝0.007）, and cytogenetic high risk（*HR*＝3.69, 95％ *CI*: 1.85–7.37, *P*<0.001）as independent risk factors affecting the PFS of patients. Meanwhile, CPC of ≥0.105％（*HR*＝3.50, 95％ *CI*: 1.29–9.48, *P*＝0.014）and positive CPC after induction therapy（*HR*＝4.12, 95％ *CI*: 1.13–15.03, *P*＝0.032）are independent risk factors affecting the OS of patients.

**Conclusion:**

Patients with NDMM demonstrating high CPC expression have a worse prognosis, with CPC level as an independent prognostic factor.

多发性骨髓瘤（MM）是临床上常见的血液系统恶性肿瘤，其特征是骨髓中存在异常增生的单克隆浆细胞。单克隆浆细胞不仅局限于骨髓，随着病情的进展，可进入血液循环，称为循环浆细胞（CPC），CPC与MM患者的预后密切相关[1]。在MM的诊治过程中，CPC作为一项关键的生物标志物已受到越来越多的重视，中华医学会血液学分会浆细胞疾病学组及实验诊断学组制定了相应专家共识[2]。

本研究对我院接受VRD方案（硼替佐米+来那度胺+地塞米松）诱导治疗的初诊MM（NDMM）患者进行回顾性分析，旨在探讨CPC在目前VRD方案标准化治疗下对MM患者预后的预测效能，并评估其在临床治疗中作为潜在生物标志物的应用价值。通过深入分析CPC与患者预后的关系，为MM患者的个性化治疗提供更加精准的指导。

## 病例与方法

1. 病例：回顾性分析2019年1月至2024年3月江苏省人民医院血液科诊治的152例接受VRD方案治疗的NDMM患者，排除确诊后未规范应用VRD方案诱导治疗及资料不全的患者。MM的诊断标准参照《中国多发性骨髓瘤诊治指南（2022年修订）》[3]。本研究获得南京医科大学第一附属医院伦理委员会批准（批件号：2023-SR-56）。

2. 临床资料：收集患者性别、年龄、血常规、血肌酐、血清白蛋白定量、LDH、β_2_-微球蛋白（β_2_-MG）、免疫固定电泳、疾病分期与分层、治疗方案、初诊及治疗后CPC比例、疗效评估、细胞遗传学检查结果等。

3. 治疗方案：152例患者均接受至少3个疗程的VRD方案［硼替佐米1.3 mg/m^2^，皮下注射，第1、4、8、11天或第1、8、15、22天；来那度胺25 mg（根据肌酐清除率调整剂量）口服，第1～15天；地塞米松20 mg，静脉或口服，第1～2、4～5、8～9、11～12天，年龄>75岁时地塞米松剂量减半］诱导治疗，其中69例患者在至少4个周期的诱导治疗后接受自体造血干细胞移植（auto-HSCT），2例患者接受CAR-T细胞治疗。

4. 疗效评估：疗效评估依据国际骨髓瘤工作组（IMWG）2016年制定的疗效标准。主要评估指标包括无进展生存（PFS）、总生存（OS）。次要评估指标为治疗后疗效，分为严格意义的完全缓解（sCR）、完全缓解（CR）、非常好的部分缓解（VGPR）、部分缓解（PR）、疾病稳定（SD）和疾病进展（PD），其中客观缓解率（ORR）定义为sCR率、CR率、VGPR率和PR率之和，微小残留病灶（MRD）阴性定义为疗效评估达CR及以上，且应用二代流式细胞术检测10^6^个骨髓有核细胞，未检测到异常克隆性浆细胞。

5. 随访：通过查阅门诊、住院病历及电话进行随访，随访截止日期为2024年6月30日，中位随访时间为31（3～64）个月。PFS期定义为自患者确诊至疾病进展、复发或死亡的时间，OS期定义为自患者确诊至死亡或随访截止的时间。

6. 流式细胞术检测CPC：通过8色流式细胞术检测CPC，使用以EDTA作为抗凝剂的试管收集血液样本，并在收集后24 h内通过流式细胞术进行评估，取适量外周血加入流式检测管（根据全血细胞计数进行调整，保证每管有核细胞总数>10^6^个），一次性加入特异性抗体，特异性抗体购自美国BD公司。根据CD38^+^、CD138^+^表达圈定总浆细胞。CD19^+^CD56^-^细胞识别为正常浆细胞（nPC），CD19^+^CD56^+^、CD19^-^CD56^+^和CD19^-^CD56^-^细胞识别为异常浆细胞（aPC）。然后分析了aPC和nPC的cκ/cλ比值，根据胞质轻链比值（κ∶λ>3.0或<0.3）对CPC进行鉴定。每管至少分析500 000个有核细胞。CPC比例为CPC计数/血液中总有核细胞计数。CPC阴性定义为血液中无CPC，检测下限为1×10^−4^，CPC阳性定义为CPC高于此阈值。

7. 统计学处理：SPSS 25.0和GraphPad Prism 8.0软件进行统计学分析。计量资料采用*x*±*s*表示，计数资料采用例数（％）表示。患者临床特征的比较采用Mann Whitney *U*检验、*χ*^2^检验。采用Kaplan-Meier法绘制生存曲线，显著性检验采用Log-rank法。多因素分析采用Cox比例风险模型。*P*<0.05为差异有统计学意义。

## 结果

1. 临床特征：152例患者中男76例（50％），女76例（50％），中位发病年龄62（40～77）岁。107例（70.4％）患者检出CPC，45例（29.6％）未检出CPC。根据ROC分析，预测OS的最佳截断值为0.105％，敏感度为47.4％，特异度为77.4％。据此将患者分为两组：CPC比例≥0.105％组39例（25.7％），CPC比例<0.105％组113例（74.3％）。比较两组患者的临床特征，与CPC比例<0.105％组相比，CPC比例≥0.105％组患者初诊时β_2_-MG≥3.5 g/L比例（*P*<0.001），HGB≤100 g/L比例（*P*＝0.015），ISS分期Ⅲ期比例（*P*<0.001）和R-ISS分期Ⅲ期比例（*P*＝0.023）更高（表1）。

**表1:** CPC比例<0.105％与≥0.105％组多发性骨髓瘤患者的临床特征比较

临床特征	CPC比例<0.105％（113例）	CPC比例≥0.105％（39例）	*χ*^2^值/*OR*值	*P*值
性别［例（％）］			0.310	0.577
男	58（51.3）	18（46.2）		
女	55（48.7）	21（53.8）		
年龄［例（％）］			0.353	0.553
≥65岁	46（40.7）	18（46.2）		
<65岁	67（59.3）	21（53.8）		
分型［例（％）］				
IgG	59（52.2）	17（43.6）		
IgA	37（32.8）	8（20.5）		
轻链型	17（15.1）	9（23.1）		
其他	1（0.9）	4（10.3）		
β_2_-微球蛋白			13.694	<0.001
<3.5 g/L	56（50.0）	6（15.8）		
≥3.5 g/L	56（50.0）	32（84.2）		
白蛋白			0.099	0.753
<35 g/L	67（59.3）	22（56.4）		
≥35 g/L	46（40.7）	17（43.6）		
肌酐			0.001	0.969
<177 µmol/L	109（97.3）	38（97.4）		
≥177 µmol/L	3（2.7）	1（2.6）		
乳酸脱氢酶			0.016	0.899
≤271 U/L	105（92.9）	36（92.3）		
>271 U/L	8（7.1）	3（7.7）		
血红蛋白			5.903	0.015
>100 g/L	51（45.1）	9（23.1）		
≤100 g/L	62（54.9）	30（76.9）		
血小板计数			1.424	0.233
≥100×10^9^/L	101（89.4）	32（82.1）		
<100×10^9^/L	12（10.6）	7（17.9）		
DS分期［例（％）］			3.722	0.156
Ⅰ期	4（3.5）	0（0）		
Ⅱ期	23（20.4）	4（10.3）		
Ⅲ期	86（76.1）	35（89.7）		
ISS分期［例（％）］			15.719	<0.001
Ⅰ期	32（28.3）	4（10.3）		
Ⅱ期	57（50.4）	14（35.9）		
Ⅲ期	24（21.2）	21（53.8）		
R-ISS分期［例（％）］			7.511	0.023
Ⅰ期	24（21.8）	2（5.1）		
Ⅱ期	79（71.8）	31（79.5）		
Ⅲ期	7（6.4）	6（15.4）		
细胞遗传学［例（％）］				
高危	22（19.5）	9（23.1）	0.232	0.630
P53缺失	9（8.5）	5（12.8）	0.613	0.434
1q21扩增	53（50.0）	18（46.2）	0.169	0.681
t（4;14）	15（13.3）	4（10.3）	1.927	0.251
t（14;16）	1（0.9）	0（0）	1.053	0.263
≥2个高危细胞遗传学	17（15.0）	7（17.9）	0.184	0.668
auto-HSCT［例（％）］			0.589	0.443
是	54（47.8）	15（40.5）		
否	59（52.2）	22（59.5）		

**注** CPC：循环浆细胞；auto-HSCT：自体造血干细胞移植

2. CPC与疗效的相关性分析：共146例患者可评价疗效，最佳疗效评估：ORR为97.3％，初诊时CPC比例<0.105％患者的ORR为98.2％，CPC比例≥1.105％患者的ORR为94.4％，两组患者的具体疗效见表2。

**表2:** CPC比例<0.105％与≥0.105％组多发性骨髓瘤患者的疗效比较［例（％）］

疗效	CPC比例<0.105％（110例）	CPC比例≥0.105％（36例）	*χ*^2^值/*OR*值	*P*值
MRD阴性	57（51.8）	14（38.9）	1.815	0.178
sCR	51（46.4）	15（41.7）	0.242	0.623
CR	11（10.0）	4（11.1）	0.036	0.849
VGPR	32（29.1）	12（33.3）	0.232	0.630
PR	14（12.7）	3（8.3）	0.509	0.476
SD	0（0）	1（2.8）	0	0.330
PD	2（1.8）	1（2.8）	0.648	0.642

**注** CPC：循环浆细胞；MRD：微小残留病；sCR：严格意义的完全缓解；CR：完全缓解；VGPR：非常好的部分缓解；PR：部分缓解；SD：疾病稳定；PD：疾病进展

3. 生存分析：总体患者的中位OS期未达到，中位PFS期为46.0（95％*CI*：35.57～56.43）个月。CPC比例<0.105％组患者中位OS期未达到，CPC比例≥0.105％组患者的中位OS期为52.0（95％*CI*：22.61～81.39）个月（*P*＝0.005）。CPC比例<0.105％组和CPC比例≥0.105％组患者的中位PFS期分别是54.0（95％*CI*：39.51～68.49）个月和24.0（95％*CI*：14.14～33.86）个月（*P*<0.001）（图1）。

**图1:**
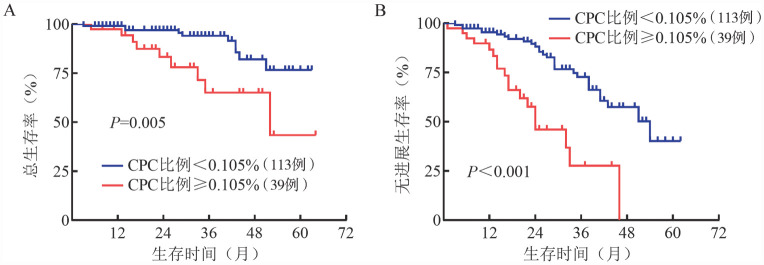
循环浆细胞（CPC）比例<0.105％和≥0.105％组多发性骨髓瘤患者的总生存（A）和无进展生存（B）曲线

4. 诱导治疗前后CPC动态变化与生存的相关性：根据MM患者初诊时CPC水平及诱导治疗4个疗程后的CPC水平分组，其中初诊和诱导治疗后CPC均为阳性（CPC阳性组）患者13例（8.5％），初诊时CPC阳性，诱导治疗后CPC阴性（CPC转阴组）患者94例（61.8％），初诊和诱导治疗后CPC均为阴性（CPC阴性组）患者45例（29.6％）。CPC阳性组的中位OS期（26个月对未达到对未达到，*P*<0.001）和中位PFS期（19个月对41个月对未达到，*P*<0.001）较CPC转阴组和CPC阴性组更短（图2）。

**图2:**
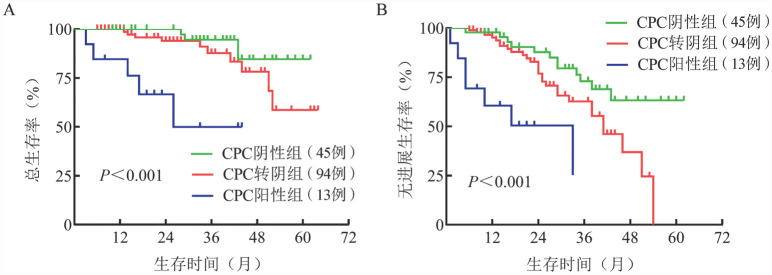
循环浆细胞（CPC）阴性组、CPC转阴组、CPC阳性组多发性骨髓瘤患者的总生存（A）和无进展生存（B）曲线

5. 单因素和多因素分析：对152例MM患者的预后进行单因素分析，纳入因素包括初诊时CPC比例、诱导治疗后CPC比例、年龄、ISS分期、R-ISS分期、HGB、PLT、白蛋白、LDH、β_2_-MG、高危细胞遗传学和是否接受auto-HSCT。随后，纳入单因素分析中*P*<0.05的影响因素，对相关因素进行筛选后行多因素分析。结果显示，影响患者PFS的独立危险因素包括CPC比例≥0.105％（*HR*＝3.79，95％ *CI*：1.95～7.38，*P*<0.001）、诱导治疗后CPC阳性（*HR*＝3.54，95％*CI*：1.41～8.87，*P*＝0.007）和高危细胞遗传学（*HR*＝3.69，95％*CI*：1.85～7.37，*P*<0.001），影响患者OS的独立危险因素包括CPC比例≥0.105％（*HR*＝3.50，95％*CI*：1.29～9.48，*P*＝0.014）和诱导治疗后CPC阳性（*HR*＝4.12，95％*CI*：1.13～15.03，*P*＝0.032）（表3）。

**表3:** 影响多发性骨髓瘤患者总生存和无进展生存的单因素、多因素分析

因素	总生存				无进展生存			
	单因素分析		多因素分析		单因素分析		多因素分析	
	*HR*（95％*CI*）	*P*值	*HR*（95％*CI*）	*P*值	*HR*（95％*CI*）	*P*值	*HR*（95％*CI*）	*P*值
年龄≥65岁	1.57（0.63～3.92）	0.331			1.05（0.58～1.88）	0.882		
CPC比例≥0.105％	3.39（1.37～8.37）	0.005	3.50（1.29～9.48）	0.014	3.84（2.07～7.12）	<0.001	4.30（2.23～8.29）	<0.001
诱导治疗后CPC阳性	6.58（2.08～20.88）	0.001	4.12（1.13～15.03）	0.032	4.59（1.90～11.11）	<0.001	3.61（1.46～8.96）	0.006
ISS分期Ⅲ期	1.27（0.48～3.36）	0.624			1.24（0.66～2.34）	0.500		
R-ISS分期Ⅲ期	1.25（0.29～5.43）	0.085			1.74（0.68～4.44）	0.247		
高危细胞遗传学	1.64（0.59～4.58）	0.342			2.20（1.19～4.30）	0.013	3.13（1.59～6.15）	<0.001
HGB≤100 g/L	0.53（0.21～1.32）	0.175			1.43（0.79～2.58）	0.237		
PLT<100×10^9^/L	0.67（0.15～2.91）	0.592			1.17（0.52～2.63）	0.707		
β_2_-微球蛋白≥3.5 g/L	1.43（0.56～3.64）	0.455			1.24（0.68～2.24）	0.485		
LDH>271 U/L	0.53（0.07～3.97）	0.534			0.96（0.34～2.69）	0.940		
白蛋白<35 g/L	0.86（0.35～2.13）	0.746			1.12（0.63～2.00）	0.698		
接受auto-HSCT	0.41（0.16～1.08）	0.071			0.59（0.33～1.07）	0.081		

**注** CPC：循环浆细胞；LDH：乳酸脱氢酶；auto-HSCT：自体造血干细胞移植

## 讨论

流式细胞术检测CPC为NDMM患者的早期诊断及预后评估提供了一种无创检查方法。既往文献报道，55％～93％的NDMM患者初诊时可在外周血检测到CPC。Gonsalves等[4]的研究纳入566例NDMM患者，其中383例（67％）患者的外周血中可检测到CPC。Cheng等[5]的研究纳入191例NDMM患者，113例（59.2％）可检测到CPC。本中心纳入152例接受VRD方案诱导治疗的NDMM患者，107例（70.4％）患者在初诊时检测出CPC，检出率与其他研究组相近。根据ROC分析，预测OS的最佳截断值为0.105％。由于各中心实验室条件、检测手段、治疗方案等不同，各中心截断值差异较大，本中心此次应用的截断值与本中心既往研究一致。CPC反映了骨髓瘤细胞的负荷及增殖活性，蒋元强等[6]的研究显示，初诊时CPC阳性和阴性组MM患者的β_2_-MG水平、白蛋白水平、肾功能检查结果有明显差异。本研究依据截断值将NDMM患者分为CPC比例<0.105％组（113例）和CPC比例≥0.105％组（39例），发现CPC比例≥0.105％组患者β_2_-MG≥ 3.5 g/L、HGB≤100 g/L、ISS分期Ⅲ期、R-ISS分期Ⅲ期比例更高，证明CPC与β_2_-MG水平和肿瘤分期相关，但未发现CPC与肾功能的相关性，考虑与硼替佐米和来那度胺使肾功能不全患者的疗效明显改善相关[7]。

CPC的形成机制尚不明确，可能涉及肿瘤微环境和细胞遗传学异常。美国梅奥诊所的一项研究表明，在NDMM患者中，t（11;14）、t（14;16）和17p缺失患者CPC水平更高[8]。An等[9]的研究纳入757例NDMM患者，结果显示，CPC水平高的患者13q缺失和t（4;14）发生率明显增高。本研究比较两组MM患者的细胞遗传学异常，结果显示，CPC比例≥0.105％组患者IgH重排的发生率高于CPC比例<0.105％组（41.0％对29.2％），但差异无统计学意义，两组患者P53缺失、1q21扩增、t（4;14）、t（14;16）的发生率无明显差异，但尚需更多临床病例验证。

对于NDMM患者，CPC水平高预示缓解深度较低、预后较差[2]。Tembhare等[10]在化疗3个周期和6个周期结束时对未接受auto-HSCT治疗的MM患者进行CPC检测，发现CPC阳性与PFS和OS相关；化疗早期CPC转阴患者具有较好的PFS，而CPC持续阳性则预示OS较差。Chakraborty等[11]的研究表明，初诊时CPC阳性患者诱导治疗后CPC转阴与移植后PFS和OS显著改善相关。本研究在患者诱导治疗4个周期后测定CPC水平，根据初诊及治疗后的CPC水平将患者分为三组，结果显示，初诊和诱导治疗后CPC持续阳性患者预后最差，治疗后CPC转阴患者的OS及PFS劣于初诊及治疗后均阴性患者，提示应持续检测NDMM患者的CPC水平，以动态评估患者的预后。

已有多项研究表明，CPC水平高是影响预后的独立危险因素[12]–[14]，但关于CPC水平对接受不同化疗方案NDMM患者预后影响的研究较少。Han等[12]的研究表明，在接受以硼替佐米为基础化疗方案的MM患者中，CPC水平高患者的预后明显劣于CPC水平低的患者。但在以免疫抑制剂为基础化疗方案的MM患者中未发现CPC水平对预后的影响。VRD方案对CPC的影响及其与患者预后的相关性值得进一步探讨。本研究针对接受VRD方案诱导治疗的NDMM患者，避免了化疗方案对NDMM患者生存的影响，结果表明，CPC水平仍是影响患者预后的独立危险因素，需要进一步强化诱导治疗方案，争取使CPC由阳性转为阴性，改善患者的PFS和OS。

综上所述，本研究表明，对于接受VRD方案诱导治疗的NDMM患者，CPC水平仍然是预后的独立危险因素，临床中应常规检测并动态观察CPC变化，以协助判断预后并及时调整治疗方案。

## References

[1] Rodriguez-Otero P, Paiva B, San-Miguel JF (2021). Roadmap to cure multiple myeloma[J]. Cancer Treat Rev.

[2] 中华医学会血液学分会浆细胞疾病学组, 中华医学会血液学分会实验诊断学组 (2024). 外周血循环浆细胞流式细胞术检测在浆细胞病中的应用中国专家共识(2024年版)[J]. 中华血液学杂志.

[3] 中国医师协会血液科医师分会, 中华医学会血液学分会 (2022). 中国多发性骨髓瘤诊治指南(2022年修订)[J]. 中华内科杂志.

[4] Gonsalves WI, Rajkumar SV, Dispenzieri A (2017). Quantification of circulating clonal plasma cells via multiparametric flow cytometry identifies patients with smoldering multiple myeloma at high risk of progression[J]. Leukemia.

[5] Cheng Q, Cai L, Zhang Y (2021). Circulating plasma cells as a biomarker to predict newly diagnosed multiple myeloma prognosis: developing nomogram prognostic models[J]. Front Oncol.

[6] 蒋 元强, 李 建勇, 吴 雨洁 (2006). 多发性骨髓瘤患者外周血与骨髓中瘤细胞的检测及临床意义[J]. 中国实验血液学杂志.

[7] Ludwig H, Drach J, Graf H (2007). Reversal of acute renal failure by bortezomib-based chemotherapy in patients with multiple myeloma[J]. Haematologica.

[8] Gonsalves WI, Jevremovic D, Nandakumar B (2020). Enhancing the R-ISS classification of newly diagnosed multiple myeloma by quantifying circulating clonal plasma cells[J]. Am J Hematol.

[9] An G, Qin X, Acharya C (2015). Multiple myeloma patients with low proportion of circulating plasma cells had similar survival with primary plasma cell leukemia patients[J]. Ann Hematol.

[10] Tembhare PR, Sriram H, Khanka T (2022). Circulating clonal plasma cells at diagnosis and peripheral blood measurable residual disease assessment provide powerful prognostication biomarkers in newly-diagnosed multiple myeloma patients treated without autologous transplant[J]. Blood.

[11] Chakraborty R, Muchtar E, Kumar SK (2017). Serial measurements of circulating plasma cells before and after induction therapy have an independent prognostic impact in patients with multiple myeloma undergoing upfront autologous transplantation[J]. Haematologica.

[12] Han W, Jin Y, Xu M (2021). Prognostic value of circulating clonal plasma cells in newly diagnosed multiple myeloma[J]. Hematology.

[13] Nowakowski GS, Witzig TE, Dingli D (2005). Circulating plasma cells detected by flow cytometry as a predictor of survival in 302 patients with newly diagnosed multiple myeloma[J]. Blood.

[14] Xia Y, Shen N, Zhang R (2023). High-risk multiple myeloma predicted by circulating plasma cells and its genetic characteristics[J]. Front Oncol.

